# Oocyte transport against fluid flow to the fertilization site in mice: contributions of cilia beating and peristalsis^†^

**DOI:** 10.1093/biolre/ioaf139

**Published:** 2025-06-25

**Authors:** Toshiaki Hino

**Affiliations:** Department of Biological Sciences, Asahikawa Medical University, Asahikawa, Hokkaido, Japan

**Keywords:** infundibulum, ampulla, oocyte transport, fluid flow, fluid production, cilia, peristalsis, lumina structure

## Abstract

The transport of ovulated oocytes to the site of fertilization involves two main processes, the initial collection of the oocyte by the fimbria and its subsequent transport through the upper ampulla. These are crucial events preceding fertilization. Around ovulation, the oviduct exhibits active fluid secretion, peristaltic movements, and ciliary beating, all of which are believed to be involved in oocyte transport. However, their specific contributions require further clarification. In this study, we investigated how these three factors influence oocyte transport to the fertilization site in the oviduct in vivo. The oviduct of anesthetized mice was installed in a fluid-circulating chamber. By introducing fixed and stained cumulus-oocyte complexes (COCs) into the fimbria and injecting a small amount of ink into the oviduct lumen, we monitored oocyte transport and fluid dynamics. Interestingly, while oviduct fluid flowed toward the fimbria, the COC moved in the opposite direction to reach the site of fertilization. Inhibiting ciliary beating disrupted both the collection (or “pickup”) and transport of the oocyte, whereas inhibiting peristalsis had no immediate impact on these processes. However, extended inhibition of peristalsis resulted in impaired oocyte transport. Under these conditions, fluid accumulated, and the oviduct lumen expanded, disrupting the intimate contact between the COC and the cilia. These findings indicate that ciliary beating, rather than fluid flow or peristalsis, propels the COC against the fluid flow toward the fertilization site. In addition, peristalsis maintains the luminal conditions required for effective transmission of ciliary propulsion to the COC.

## Teaser

Cilia activity plays a central role in oocyte transport by moving cumulus-oocyte complexes against opposing fluid flow, with peristalsis maintaining the optimal oviduct environment for successful transport.

## Graphical Abstract

**Graphical Abstract:**
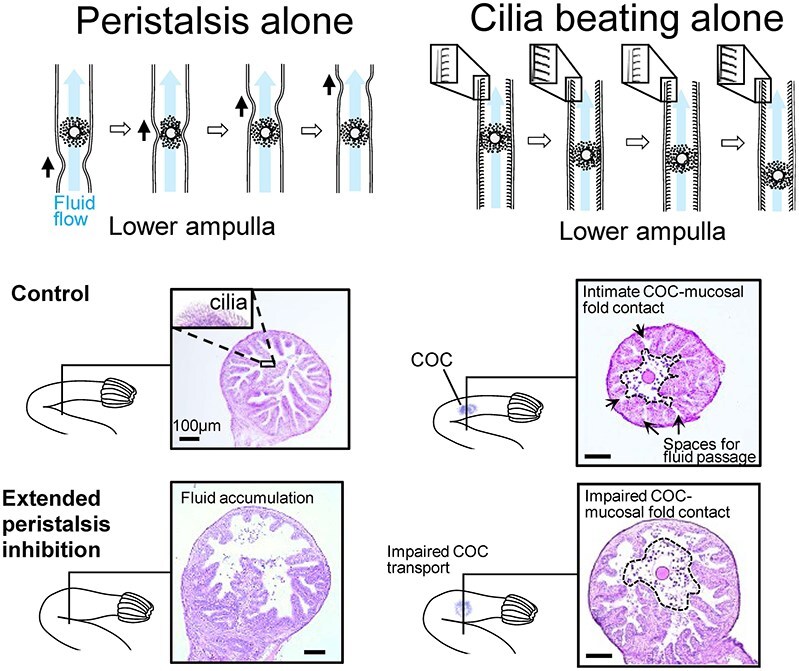

## Introduction

Oviducts are paired connecting tubes between the ovary and the uterus. Although oviductal morphology varies among species, its function remains conserved across mammals. The oviduct plays a crucial role at the beginning of life by transporting the oocyte and spermatozoa to the site of fertilization, providing an optimal environment for fertilization, and facilitating the transport of fertilized eggs to the uterus, where embryonic development continues.

Mammalian oviducts actively secrete fluid into the lumen [1, 2]. In mice and hamsters, during the estrus period, fluid is secreted along the entire length of the oviduct and propelled toward the ovary by oviductal peristalsis, generating a rapid flow within the lumen [3, 4]. We have previously demonstrated that spermatozoa in mice are transported from the lower isthmus to the fertilization site by fluid flow [4].

Given that the oocyte is ovulated and transported to the fertilization site during the estrous period, an important question arises: Can the cumulus-oocyte complex (COC) progress against the fluid flow? Although COCs can probably move contrary to fluid movement, this phenomenon has not been directly demonstrated. In addition, if this assumption holds, what factors enable the COC to move against fluid flow?

The transport of the ovulated oocyte involves two primary processes: pickup by the fimbria and transport through the upper ampulla to the fertilization site [5] (see Supplementary Figure 1 for an anatomical diagram of the mouse oviduct). The factors contributing to COC pickup and transport are believed to include oviductal muscle contractions and the activity of ciliated epithelial cells, which densely populate the fimbria and ampulla [6]. Prior research indicates that ciliary beating, rather than oviductal peristalsis, is the primary factor involved in COC pickup. In female mice, impaired ciliary motion prevented fimbrial pickup of COCs, underscoring its essential role [7, 8]. By contrast, inhibiting muscle contractions did not prevent fimbrial capture of COCs in rabbits and rats [9–11].

The transport of COCs through the upper ampulla to the fertilization site is thought to involve both ciliary activity and muscle contractions. However, this remains a topic of debate. Some studies suggest that COCs can be transported solely by ciliary motion, even in the absence of oviductal peristalsis [9–11], whereas others report that COCs fail to progress when peristalsis is inhibited, implying a critical role for muscle contractions [12, 13]. Notably, existing studies have not examined the effects of selectively inhibiting ciliary motion alone on COC transport. To determine the relative contributions of ciliary activity and peristalsis in the upper ampulla, it is essential to investigate how transport is affected when one mechanism is disrupted while the other remains active. This aspect has yet to be systematically addressed.

In the present study, we simultaneously visualized tubal fluid flow and COC movement in mouse oviducts in vivo. We tracked both behaviors to verify whether COCs progress against fluid flow to reach the fertilization site. Injecting a small bolus of ink into the oviduct allowed us to visualize fluid flow dynamics [4]. Furthermore, utilizing COCs fixed with glutaraldehyde and stained with hematoxylin enhances their visibility through the oviduct wall, allowing precise movement tracking. Employing this method, we examined the effects of selectively inhibiting either ciliary beating or peristalsis on COC transport, providing new insights into their respective contributions.

## Materials and methods

### Animals

ICR female mice aged 7 weeks were purchased from SLC Inc. (Shizuoka, Japan) and housed for 2 weeks under specific pathogen-free conditions with controlled light, temperature, and humidity (light between 7:00 and 19:00, 22 ± 2°C, 50 ± 10% humidity) before use. Female mice were injected intraperitoneally with equine chorionic gonadotropin (eCG), followed by human chorionic gonadotropin (hCG) injection (Aska Pharmaceutical) 48 h later. Specifically, mice used for COCs collection received 5.0 IU of both eCG and hCG, while mice used for the in vivo experiments on COC pickup and transport received 2.5 IU of both eCG and hCG. Because ovulation begins at approximately 11 h after hCG injection and is complete around 14 h after hCG injection [14, 15], females 14–15 h after hCG injection were used for COCs collection, and those 10–11 h after hCG injection were used for the in vivo experiments on COC pickup and transport. All animal experiments were performed in accordance with the Asahikawa Medical University Animal Experiments Guidelines.

### Chemicals

Chemicals were purchased from Nacalai Tesuque (Kyoto, Japan) unless otherwise stated. The medium used for oocyte preparation and observation of cilia beating in vitro was a bicarbonate-buffered Toyoda-Yokoyama-Hosi (TYH) medium [16]. The medium consisted of 119.37 mM NaCl, 4.79 mM KCl, 1.71 mM CaCl_2_, 1.19 mM KH_2_PO_4_, 1.19 mM MgSO_4_, 25.07 mM NaHCO_3_, 5.56 mM glucose, 1.00 mM sodium pyruvate, 4 mg/mL bovine serum albumin (BSA; AlbuMax; GibcoBRL, Auckland, New Zealand), 50 μg/mL streptomycin sulfate, and 75 μg/mL penicillin G potassium, pH 7.4 when equilibrated with 5% (v/v) CO_2_ in air. The medium for perfusing an exposed ovary-oviduct-uterus complex was Dulbecco Ca^2+^- and Mg^2+^-containing phosphate-buffered saline (PBS+) supplemented with 0.1% BSA (Sigma-Aldrich, St. Louis, MO, USA) (BSA/PBS).

### Oocyte preparation

Fourteen to 15 h after hCG injection, a clot of COCs was retrieved from the oviducts, cultured for 1 h in TYH medium, and pipetted to separate them into individual COCs. The COCs were fixed in 2% glutaraldehyde in PBS for 1 h and treated overnight with 1 M glycine in water. The COCs were then washed three times in BSA/PBS and stored in BSA/PBS at 4°C until use in experiments. Prior to use, the fixed COCs were stained with hematoxylin for 3 min and washed three times with BSA/PBS solution.

### Observation of the COC and fluid flow in the oviduct in vivo

**Figure 1:**
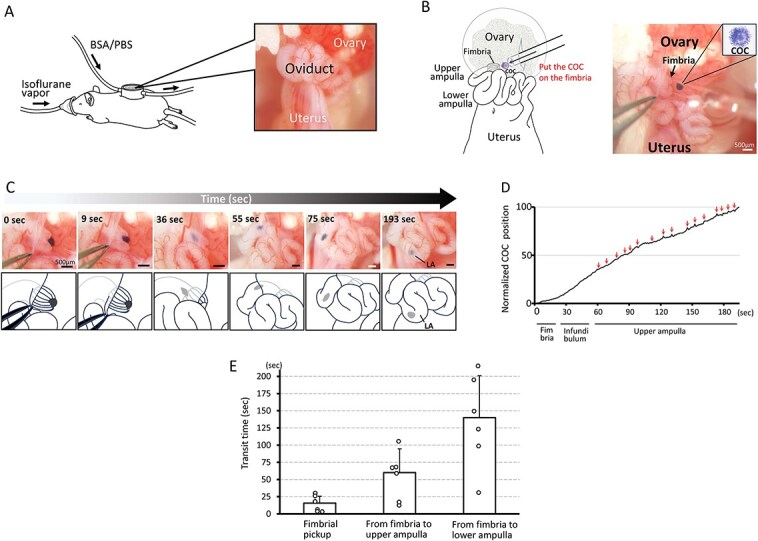
Real-time in vivo observation of the oviduct using a buffer-circulating observation chamber and tracking the pickup and transport of the COC through the infundibulum and the upper ampulla. (A) The oviduct was placed in a chamber where prewarmed buffer (BSA/PBS) was circulated, allowing real-time observation. (B) A COC, fixed with glutaraldehyde and stained with hematoxylin, was introduced onto the fimbria through a slit made in the bursa membrane. (C) Progression of the COC toward the lower ampulla. The COC was captured and drawn into the oviduct ostium at the fimbria (0–36 s), during which its shape changed from circular to oval. Maintaining its oval form, it advanced toward the lower ampulla (LA, fertilization site). (D) A trajectory graph illustrating the COC’s movement within the oviduct over time and distance. The trajectory graph was generated using still images extracted from Video 1 at a rate of 1 frame per sec. The horizontal axis represents time, while the vertical axis displays the COC’s position as a percentage—0% at the starting point (fimbria) and 100% at the endpoint (entrance of the lower ampulla). Arrows indicate when the COC displayed pendulum-like motion induced by oviduct peristalsis. Note that the trajectory graph is generated solely when the entire oviduct—from the fimbria to the lower ampulla—is aligned horizontally. Due to inherent variations in oviduct morphology, such alignment is uncommon. (E) Time required for COC pickup and transport events, shown as bar graphs with overlaid individual data points. Time is measured from the start of fimbrial pickup to its completion and the arrival at both the upper ampulla and the lower ampulla. Individual data points from each experiment are shown to illustrate data variability and distribution. Experiments were replicated six times for each condition using six animals per condition. Mean ± SD.

Behaviors of the COC and fluid flow within the oviduct were observed according to the method of Hino and Yanagimachi [4]. Female mice were anesthetized with 2% isoflurane vapor 10–11 h after hCG injection. The back skin and muscle layer were incised. The ovary and oviduct were exposed from the incision and retained on the muscle layer using a surgical adhesive (Aron Alpha A; Toagosei, Tokyo, Japan). A circulation collar (made from 15 mm inner diameter tubing) was implanted under the skin, and warmed BSA/PBS maintained at 37°C was infused into the collar to immerse the ovary (see Figure 1A).

The fixed and stained COC was aspirated with a glass capillary, the tip of the glass capillary was inserted through the slit made in the bursa membrane, and the COC was gently placed on the oviductal fimbria (Figure 1B). The behavior of the COC was recorded, and the time for the COC to reach the lower ampulla (the site of fertilization) was calculated. The process of the COC on the fimbria being completely drawn into the oviduct was defined as “COC pickup,” and the process of the COC within the infundibulum reaching the lower ampulla through the upper ampulla was defined as “COC transport.”

In some experiments, a small amount of India ink dialyzed against PBS+ overnight was injected into the oviduct from the lower part of the isthmus as soon as the COC passed through the fimbria.

### Inhibition of oviduct peristalsis

Nicardipine (Merck KGaA, Darmstadt, Germany) 20 μM in BSA/PBS was circulated through the circulation chamber.

### Inhibition of cilia beating

Sodium orthovanadate (Santa Cruz Biotechnology, Dallas, TX, USA), which can disrupt cilia activity by inhibiting the ATPase [17], was adjusted to 100 mM with distilled water and less than 0.1 μL was injected into the lumen of the oviduct ampulla. In some experiments, a small amount of India ink was added to the vanadate solution to visualize and follow the vanadate solution within the oviduct.

### Counting of cilia beating in vitro

The upper ampulla was carefully excised and transferred into TYH medium. A longitudinal slit was made, allowing the upper ampulla to retract naturally and expose the lumen (Supplemental Figure 2A). Because cilia are present on the inner surface of the lumen, the tissue was positioned so that the open side faced laterally. A glass slide, preloaded with four vaseline/paraffin mixtures (9:1) at each square’s four corners, was prepared, and the tissue was centered on these spots. A coverslip was gently placed and lightly pressed over the spots, ensuring the lateral-facing cilia remained free from direct contact with the coverslip and the glass slide (Supplementary Figure 2A). The cilia beating was observed under the microscope with a digital camera and a glass slide heater using a 100× objective lens equipped with a lens heater. During observation and recording, fresh TYH medium warmed at 37°C was introduced at 2-min intervals via a gap between the glass slide and the coverslip, and the old medium was aspirated with a filter paper on the other side of the gap (see Supplementary Figure 2B). In some experiments, warm orthovanadate or nicardipine solutions (37°C) were poured through the gap. The frequency of cilia beating was counted from recorded videos edited at 10× slow speed using CyberLink PowerDirector 2024 software (CyberLink Corp, Tokyo, Japan).

### Measurement of the fluid volume in the oviduct

The volume of the oviduct fluid was measured as described [4]. Briefly, the oviduct was ligated at the infundibulum and the utero-tubal junction using sterile 11-0 nylon threads (Bear Medic Corp, Ibaraki, Japan). The oviduct was isolated along the ligation threads, its surface was carefully wiped with absorbent tissue paper, and the oviduct was weighed. The oviduct was then incised on a filter paper in several places and pressed under another filter paper to remove the fluid in the oviduct, before being reweighed. The fluid volume was calculated by assuming that the fluid’s density was equivalent to distilled water (1 mg = 1 μL).

### Cryosectioning of the upper ampulla

Because some oviducts and lumen shrank due to dehydration when cryosections were prepared using the standard procedure employing 4% paraformaldehyde (PFA) and 10% to 30% sucrose, we used 0.9% PFA and 10% sucrose, which are of equal osmolarity to body fluid, to avoid dehydration of the tissues and oviduct lumen to reveal the precise morphological features and interrelationships of the lumen, mucosa, and COC within the upper ampulla.

Before removing the upper ampulla, the infundibulum and the lower ampulla were ligated to prevent leakage of luminal fluid. The upper ampulla was then excised along the ligation margins and fixed overnight in 0.9% PFA prepared in distilled water. In some experiments, while a fixed and stained COC was progressing through the upper ampulla, the infundibulum and lower ampulla were ligated to prevent leakage, and the upper ampulla was subsequently excised and fixed. The next day, the upper ampulla was immersed overnight in 10% sucrose in distilled water, then embedded in OCT compound, frozen in liquid nitrogen, and stored at −78°C. Sections of the upper ampulla (5 μm) were cut at −20°C using a cryostat (Leica CM3050S; Leica Biosystems, Tokyo, Japan), and stained with periodic acid-Schiff and hematoxylin.

### Measurement of the COC aspect ratio within the upper ampulla

To evaluate COC morphological change in the upper ampulla and explore its relationship with mucosal contact, we measured the COC’s aspect ratio. Representative still images were selected from recorded videos when the entire COC was clearly visible and sharply focused as it passed through the upper ampulla. The aspect ratio was determined by dividing the minor axis (i.e., the longest diameter perpendicular to the major axis) by the major axis (i.e., the longest diameter) of the COC in each selected image frame.

### Statistical analysis

The normality of the data distribution for the transit time of COC from the fimbria to the lower ampulla, the oviductal fluid volume, and the aspect ratio of the COC in the upper ampulla was confirmed using the Shapiro–Wilk test. Welch *t* test was applied to normally distributed data, while Mann–Whitney U test was used for data that did not follow a normal distribution, with comparisons based on the presence or absence of nicardipine treatment. Kaplan–Meier analysis was specifically performed on experiments where peristalsis was inhibited by nicardipine for 1 h. Time-to-event curves for COC progression were generated, excluding non-progressing cases. The log-rank test compared progression times. A two-tailed *P*-value of <0.05 was considered statistically significant. All statistical analyses were conducted using R software (version 4.5.0).

## Results

### COC behavior during pickup by fimbria and transport through the infundibulum and the upper ampulla to the fertilization site

When a COC was placed on the fimbria (Figure 1B and C, Video 1), it was drawn into the oviduct ostium within a short period varying from a few to a dozen sec (for a more detailed view of fimbrial pickup, see Video 2). Upon entering the oviduct ostium, the COC changed shape from a circular to an oval form (Figure 1C). The progression through the infundibulum was smooth and unidirectional.

In the upper ampulla, the COC moved steadily but began to exhibit a pendulum-like oscillatory movement at intervals of 10–20 s, corresponding with oviductal muscle contractions (Figure 1D). This oscillatory motion persisted after the COC reached the lower ampulla. Across all experiments, the COC was successfully picked up and transported to the fertilization site, with an average time for transport from the fimbria to the lower ampulla of 139 s (Table 1). Figure 1E displays the individual times for COC pickup by the fimbria, progression to the upper ampulla, and arrival at the lower ampulla, presented as bar graphs with overlaid individual data points.

**Table 1:** Time for the COC to migrate from the fimbria to the lower ampulla after oviduct peristalsis or cilia beating inhibition

Treatment^*^		No. of experiments^**^	No. of experiments in which COC reached lower ampulla	Time (s) for COC to reach lower ampulla from fimbria
Nicardipine	Orthovandate			
–	–	6	6	139 (34 to 222)
+^***^	–	5	5	117 (63 to 143)
–	+^****^	4	0	–
–	+^*****^	6	0	–

^*^+: treatment applied, −: treatment not applied

^**^The number of experiments corresponds to the number of animals used

^***^COC was put on the fimbria soon after peristalsis was stopped

^****^COC was put on to the fimbria soon after orthovanadate was injected

^*****^Orthovanadate was injected when the COC had reached the upper ampulla.

Additionally, these observed behaviors, particularly the morphological change upon entering the oviduct and the successful transport of the COC to the lower ampulla (Figure 1C), demonstrate that the fixed and stained COCs retained flexibility. This supports their validity as a model for subsequent in vivo experiments.

### COC transport through the upper ampulla in the context of fluid flow toward the ovary

**Figure 2:**
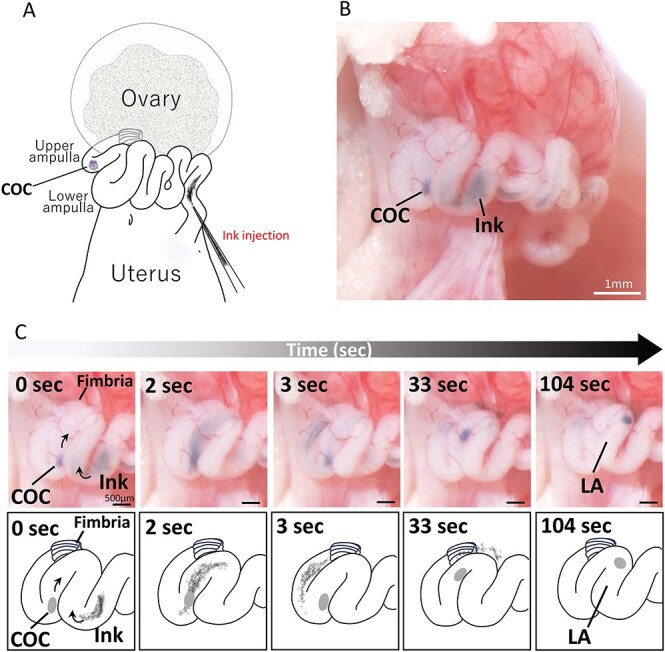
Simultaneous visualization of oviductal fluid flow and COC movement. (A) India ink was injected into the lower isthmus shortly after the COC reached the upper ampulla. (B) The ink reached the lower ampulla—the fertilization site—within 16 s of injection. (C) The intersection between the ink and the COC was observed in the upper ampulla. Notably, the COC migrated in the direction opposite to the fluid flow, and neither the fluid flow nor the COC affected or disrupted the other (Video 3 for further details). LA: lower ampulla.

To assess the interaction between fluid flow and COC transport, India ink (<0.1 μL) was injected into the lower part of the oviduct isthmus immediately after the COC reached the upper ampulla (Figure 2A, Video 3). The ink moved toward the ovary in the lower and middle isthmus while exhibiting a to-and-fro motion. Beyond the middle isthmus (Figure 2B, 16 s after ink injection), the ink moved steadily in a unidirectional flow toward the ovary, reaching the fimbria in 5 s. It then passed through the fimbria.

When the India ink and the COC intersected in the upper ampulla (Figure 2C, Video 3), the fluid flow conducting the ink was not disrupted by the presence of the COC. Moreover, the COC was not repulsed by the fluid movement and continued to move forward, eventually reaching the lower ampulla. Peristalsis in the lower and upper ampulla consistently directed the fluid toward the ovary, resulting in a unidirectional fluid flow.

### Successful COC pickup by fimbria and transport through the upper ampulla in the absence of oviduct peristalsis

**Figure 3:**
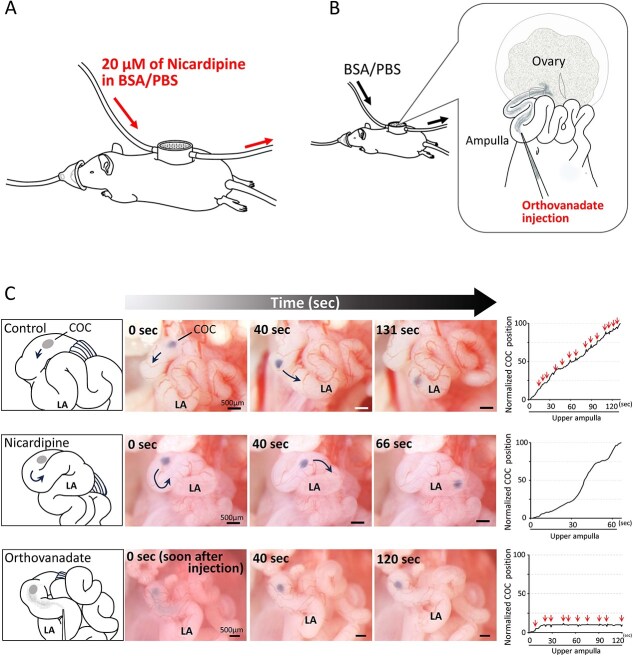
COC transport in the upper ampulla when oviduct cilia or peristalsis alone was inhibited. (A) Oviduct peristalsis was inhibited by circulating 20 μM nicardipine in BSA/PBS into the observation chamber. (B) Cilia beating was inhibited by injecting 100 mM orthovanadate solution into the lower ampulla. Injected orthovanadate was eventually expelled from the ampulla via peristalsis. (C) The behavior of the COC within the upper ampulla and its movement. The top row displays the control group—note that the oviduct in this group is identical to that depicted in Figure 1—the middle row illustrates the nicardipine-treated group, and the bottom row illustrates the orthovanadate-treated group, all demonstrating COC transport; a trajectory graph is positioned on the right. The trajectory graphs were generated by extracting still images from the videos (see Video 1 for the control oviduct, Video 4 for nicardipine-treated oviduct, Video 5 for orthovanadate-injected oviduct) at a rate of 1 frame per sec, following the approach used in Figure 1D. Note that the trajectory graphs depict the movement of COCs starting from their initial position in the upper ampulla (0-s images) toward the fertilization site (lower ampulla). Arrows indicate the time at which the COC’s pendulum motion, induced by the peristalsis of the oviduct, was observed. Notably, the COC progressed to the lower ampulla without peristalsis but failed to do so when cilia activity was inhibited. LA: lower ampulla.

**Figure 4:**
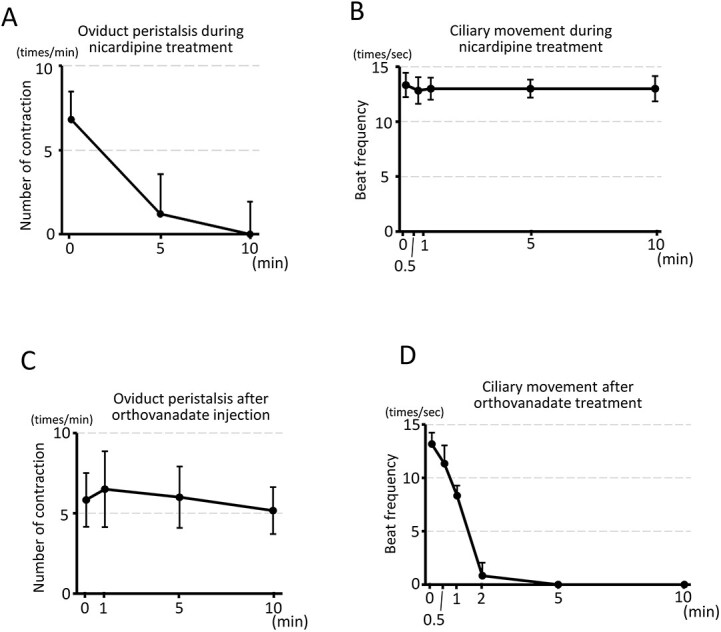
Alterations in cilia beating and peristalsis following 20 μM nicardipine or 100 mM orthovanadate treatment. Nicardipine treatment inhibited oviduct peristalsis within 10 min (A), while the beat frequency remained unaffected (B). By contrast, orthovanadate treatment did not inhibit oviduct peristalsis (C), but almost completely abolished cilia beating within 2 min (D). Note that the number of contractions was visually counted on the oviduct installed in the observation chamber (as described in Figure 1A). By contrast, the beat frequency of cilia was calculated in vitro (as described in Supplementary Figure 2). Experiments were replicated five to six times for each condition using five to six animals per condition. Mean ± SD.

To examine whether oviductal peristalsis is required for COC pickup and transport, 20 μM nicardipine in BSA/PBS was circulated in the observation chamber (Figure 3A) to inhibit oviduct muscle contraction. Approximately 10 min after nicardipine treatment, peristalsis ceased (Figure 4A). Immediately, the COC was positioned on the fimbria, where it was passively drawn into the oviduct ostium within a few to a dozen sec.

The COC that passed through the fimbria and infundibulum progressed at a slow, constant rate without any oscillatory motion in the upper ampulla (Figure 3C, Video 4). There was no significant difference in the time required for the COC to arrive at the lower ampulla between the nicardipine-treated and untreated oviducts (Table 1). In addition, no change in the cilia beat frequency was observed following nicardipine treatment (Figure 4B).

The individual times required for COC pickup by the fimbria, progression to the upper ampulla, and arrival at the lower ampulla are shown as bar graphs with overlaid individual data points in Supplemental Figure 3A.

### Complete failure of COC pickup by the fimbria and transport through the upper ampulla following inhibition of cilia beating

To determine whether cilia beating is required for COC pickup and transport, a 100 mM orthovanadate solution (<0.1 μL) was injected into the oviduct lumen to inhibit cilia activity (Figure 3B). Continuous orthovanadate treatment was not feasible due to oviduct peristalsis, which remained unaffected (Figure 4C, Video 5). The orthovanadate solution was expelled shortly thereafter, limiting the duration of ciliary exposure.

Despite the short exposure, cilia beating was inhibited, as confirmed in an in vitro experiment. Cilia treated with orthovanadate solution for 30–60 s, and then washed with TYH medium (Supplementary Figure 2B) exhibited reduced cilia beating within 30 s, which nearly completely ceased after 2 min and remained suppressed for at least 10 min (Figure 4D and Video 6).

To test whether peristalsis alone could facilitate COC transport through the upper ampulla to the fertilization site (lower ampulla), we injected an orthovanadate solution when the COC reached the upper ampulla (Figure 3C). Although the COC continued oscillating due to oviduct peristalsis, its forward progression completely ceased (Figure 3C, Video 5). The individual times required for COC pickup by the fimbria and progression to the upper ampulla are shown as bar graphs with overlaid individual data points in Supplemental Figure 3B. When the COC was placed on the fimbria after orthovanadate treatment, the fimbrial pickup of the COC was absent (Table 1).

### Impaired COC transport in the upper ampulla following extended inhibition of oviduct peristalsis

**Figure 5:**
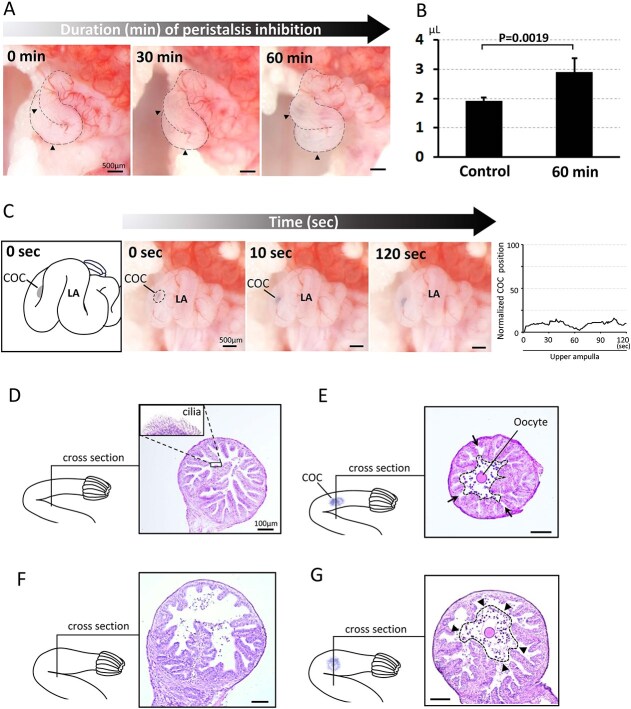
Extended inhibition of oviduct peristalsis leads to fluid accumulation, oocyte transport impairment, and oviduct luminal structure alteration. (A) Morphological changes in the upper ampulla during inhibition of peristalsis by nicardipine treatment. (B) Increase in oviductal fluid volume following 60 min of peristalsis inhibition. Experiments were replicated five times for each condition using five animals per condition. Mean ± SD. (C) Impaired COC transport within the upper ampulla after prolonged inhibition of peristalsis and its trajectory. The trajectory graph was generated by extracting still images from the video (see Video 7) at a rate of 1 frame per sec, identical to the approach utilized in Figure 3C. The COC was successfully picked up and reached the upper ampulla. However, it did not progress to the lower ampulla (LA) within the 10-min recording period. (D–G) Histology of the upper ampulla under normal and peristalsis-inhibited conditions. In untreated oviducts, the lumen of the upper ampulla was filled with mucosal folds extending toward the center (D). When a COC was present, these mucosal folds were in close contact with almost the entire surface of the COC, and open spaces were observed between the folds at the peripheral regions of the lumen (indicated by arrows) (E). By contrast, in oviducts subjected to 60 min of peristalsis inhibition with nicardipine, the lumen of the upper ampulla was markedly dilated (F). Under these conditions, when a COC was present, some areas of its surface were not in contact with the mucosal folds (arrowheads), with approximately 50% of the area remaining noncontacting (G).

Extended peristalsis inhibition visibly distended the oviduct, increasing fluid volume by 50% (Figure 5A and B). Notably, nicardipine treatment did not affect fluid production [4]. This increased fluid volume can be attributed exclusively to the inhibition of peristalsis. The fimbria successfully picked up the COC in all experiments. However, in two of six cases, the COC failed to progress beyond the upper ampulla (Figure 5C, Video 7). In the remaining four cases, the COC reached the lower ampulla, but the transit time was prolonged (229 s vs 139 s in the untreated control group). Kaplan–Meier analysis, accounting for censored data, confirmed a significant delay in transit time in the nicardipine-treated group compared with the untreated control (*P* < 0.05).

Figure 5D–G illustrates the structural differences in the upper ampulla under normal and peristalsis-inhibited conditions. In the untreated oviduct, the mucosa formed long, longitudinal folds that nearly met in the center of the lumen (Figure 5D). When a COC was present, these mucosal folds extensively contacted the cumulus matrix surface (Figure 5E). However, the COC did not fully occupy the lumen, leaving gaps between longitudinal folds at the periphery (Figure 5E, arrows). By contrast, the peristalsis-inhibited oviduct exhibited a widened lumen (Figure 5F), and the COC in the distended ampulla displayed reduced mucosal contact (Figure 5G, arrowheads).

**Figure 6:**
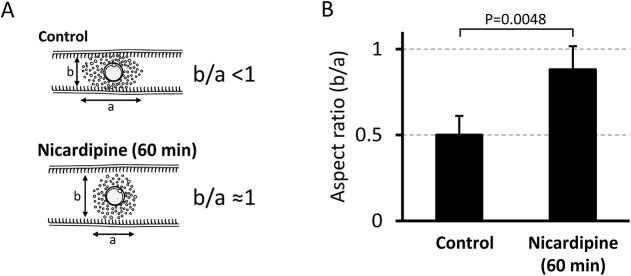
Aspect ratio of the COC in the upper ampulla for both the control and 60-min nicardipine-treated groups as an indicator of the intimate contact between the COC and the mucosal folds. (A) Illustrations of the COC in the upper ampulla in the control group and the 60-min nicardipine-treated group. In the control group, the COC exhibits an oval shape with an aspect ratio (b/a) of less than 1 (<1). In contrast, in the 60-min nicardipine-treated group, the distended upper ampulla leads the COC to change from oval to nearly circular, resulting in an aspect ratio close to 1 (≈1). Consequently, the broad and intimate contact between the COC and the mucosal folds is impaired. (B) The aspect ratio of the COC in the upper ampulla was significantly higher in the 60-min nicardipine-treated group than in the control group. Aspect ratios were calculated by extracting representative still images from recorded real-time videos (five to six videos per group). Mean ± SD.

To assess mucosal contact, we quantified the COC aspect ratio in the upper ampulla for both the control and nicardipine-treated groups. In the distended upper ampulla, the reduced mechanical constraints not only weakened the intimate contact with the mucosa (Figure 5G) but also reversed the pressure-induced deformation of the COC—returning it from an oval to its natural circular form (Figure 6A). The aspect ratio, defined as the ratio of the minor axis to the major axis, was significantly higher in the nicardipine-treated group compared to the control group (Figure 6B).

## Discussion

The present study demonstrated that the COC moved against the oviduct fluid flow to reach the fertilization site, relying predominantly on ciliary beating. Although peristalsis did not contribute directly to the force that propels the COC, our data indicate that it maintains the luminal conditions necessary for intimate contact between the COC and mucosa folds, thereby ensuring effective transmission of cilia propulsion.

Although the question of whether peristalsis, along with cilia beating, may propel the oocyte in the upper ampulla remains unanswered, our study clearly demonstrated that peristalsis does not directly provide a propulsive force in mice. This conclusion is directly supported by two key observations. First, when cilia beating was exclusively inhibited, the COC still displayed pendulum-like movements in response to peristalsis, failing to progress toward the fertilization site. Second, as reflected by the movement of India ink through the ampulla (Video 3), peristalsis around ovulation directed toward the ovary–opposite to the direction of COC progression.

Although peristalsis did not serve as the propulsive force for oocyte transport to the lower ampulla, our results indicated that peristalsis plays an important role in maintaining the luminal structure and ensuring that the COC remains in close contact with the mucosal folds, which in turn facilitates the effective transmission of ciliary propulsion. In the present study, continuous inhibition of oviduct peristalsis caused oviduct distention (Figure 5A), delaying or halting COC transport, despite successful pickup by the fimbria (Video 7, Figure 5C). Hino and Yanagimachi [4] reported that the oviduct actively secretes fluid during the estrous period, and oviduct peristalsis drives fluid efflux into the ovarian bursa, where it eventually drains into the abdominal cavity through the bursa hole. This study revealed that when the “pumping action” of peristalsis continued to be inhibited, fluid accumulated (Figure 5B), the lumen expanded (Figure 5F), and intimate contact between the COC and mucosa folds was impaired (Figure 5G). The cilia lining on mucosal folds, which act to push the COC forward, were partially separated from the COC surface (Figure 5G arrowheads), likely disrupting ciliary propulsion. Notably, fluid drainage via a small slit in the ampulla resolved the distention and allowed the COC to resume progress (data not shown).

Our observation that the COC was successfully picked up and transported to the site of fertilization without peristalsis supports observations in earlier experiments in rats and rabbits [9–11]. However, these findings contrast with Dixon et al. [12], who reported that in mice COC progression in the ampulla disappeared upon inhibition of muscle contraction. We attribute this discrepancy primarily to methodological differences because although our study and that of Halbert et al. [9–11] employed the oviduct in its in vivo state, Dixon et al. [12] utilized isolated oviducts. It is important to note that in vitro observations do not necessarily reflect in vivo conditions. Separating the oviduct from the body terminates blood supply and neural regulation and could modify oviductal function. For instance, although Miki et al. [18] reported that, in vitro, the oviduct fluid of an estrous female mouse flows toward the uterus, Battalia and Yanagimachi [3] and Hino and Yanagimachi [4] reported that in vivo it flows rapidly in the opposite direction, toward the ovary.

Interestingly, the COC continued to progress through the upper ampulla despite the oviduct fluid flow (see Figure 2C and Video 3). Histological examination revealed that the upper part of the longitudinal folds surrounded the COC, occupying the central lumen (Figure 5E); whereas by contrast, gaps existed at the periphery where the middle and lower parts of the folds did not contact the COC (Figure 5E, arrows). This luminal architecture potentially allowed the oviduct fluid to pass through the upper ampulla as the COC progressed. Analogous luminal structures of the ampulla have also been observed in many mammals, including ewes [19], bovines [20], and humans [21].

Potential off-target effects of pharmacological inhibitors, particularly on oviduct fluid secretion, must be considered when interpreting our results. Nicardipine, used for peristalsis inhibition, did not affect fluid production in a previous study [4]; moreover, the observed fluid accumulation during extended peristalsis inhibition further supports continuous secretion. In the case of sodium orthovanadate, since it was locally injected and rapidly expelled by unaffected peristalsis, exposure was limited. Although we did not directly measure its impact on fluid secretion, given that the oviduct fluid is secreted along the entire length, the overall effect on the fluid flow toward the ovary was likely minimal. Therefore, we infer that these potential off-target effects did not substantially confound our primary conclusions.

Kartagener syndrome, also known as primary ciliary dyskinesia, is a genetic human disorder characterized by sinusitis or bronchiectasis, and it is associated with oviduct cilia dyskinesia in women [22, 23]. Interestingly, some women with Kartagener syndrome are reported to be fertile [24, 25], which has been interpreted as supporting the hypothesis that peristalsis, rather than cilia activity, plays the primary role in oocyte transport to the fertilization site in humans. Notably, Halbert [26] observed that some women with complete cessation of tracheal cilia beating exhibited residual, though subnormal, oviductal cilia activity. Although we did not identify any studies directly investigating oviduct cilia motility in fertile women with Kartagener syndrome, a recent study in mice reported that females with decreased oviduct cilia motility were still capable of, albeit subfertile, becoming pregnant [8]. To clarify this discrepancy, further studies should directly assess the cilia function in the oviducts of fertile women with Kartagener syndrome.

While the current study provides compelling evidence that cilia activity is the primary factor for oocyte transport to the fertilization site in the mouse oviduct and peristalsis is required for maintaining the luminal environment crucial for effective ciliary propulsion, our conclusions must be viewed in the context of the experimental limitations. These limitations include the use of externalized oviducts immersed in buffer, which could impact physiological conditions and contractility. Future work employing comparative studies across species and direct assessments in human subjects will be pivotal in fully elucidating the precise mechanisms underlying oocyte transport.

## Supplementary Material

Supplemental_Figure_1_ioaf139

Supplemental_Figure_2_ioaf139

Supplemental_Figure_3_ioaf139

1_COCtransport_ioaf139

2_fimbrial_pickup_ioaf139

3_COC_and_Ink_intersection_ioaf139

4_COCtransport_in_the_abscence_of_peristalsis_ioaf139

5_COCtransport_in_the_abscence_of_cilia_beating_ioaf139

6_Cilia_beating_after_vanadate_treatment_ioaf139

7_COCtransport_1h_after_peristalsis_inhibition_ioaf139

## Data Availability

Data supporting the findings of this study are available from the corresponding author upon reasonable request.
