# Supplementary figures and images for: Oocyte transport against fluid flow to the fertilization site in mice: contributions of cilia beating and peristalsis
Source: Biol Reprod. 2025 Jun 25;113(3):557–67. doi: 10.1093/biolre/ioaf139 (PMC13128679; doi:10.1093/biolre/ioaf139)

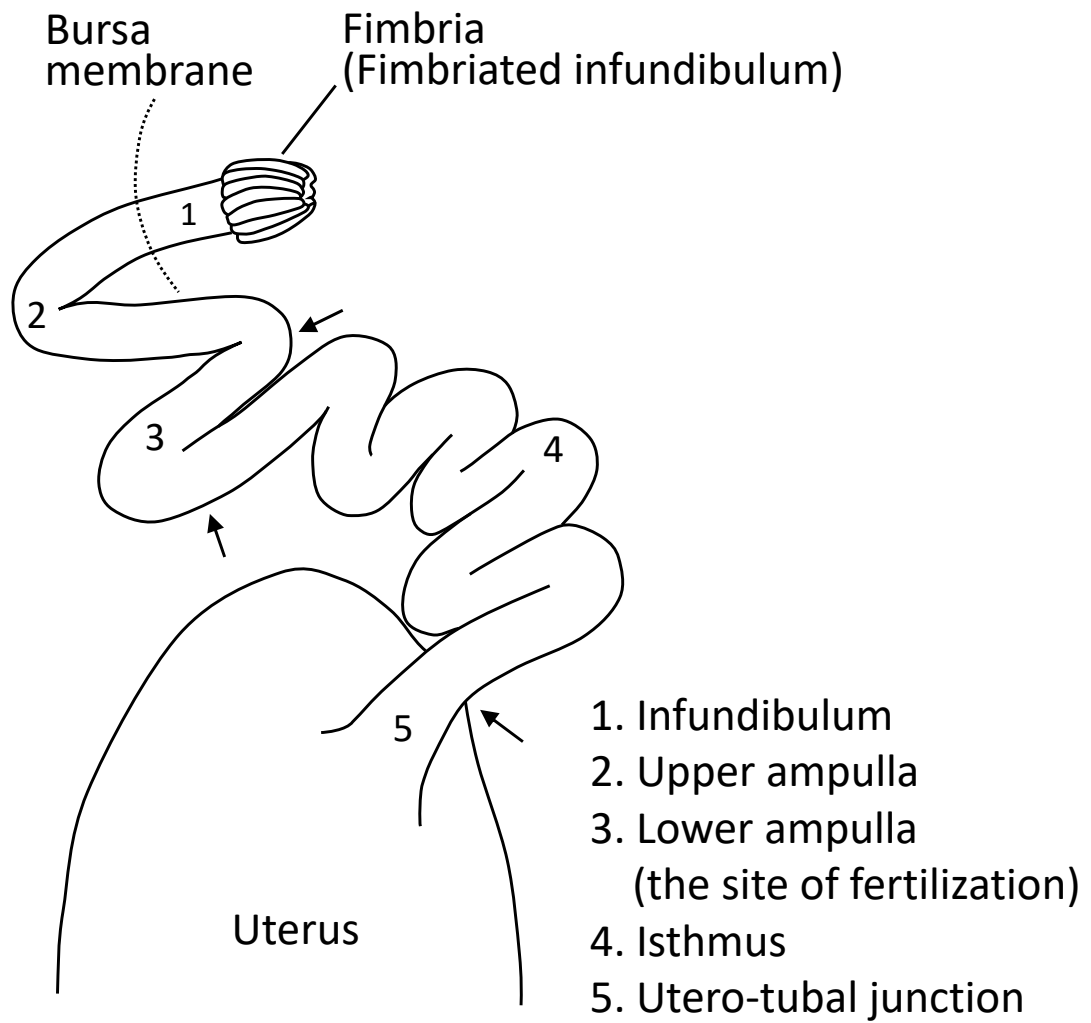

Supplement: Supplemental_Figure_1_ioaf139 [file supplemental_figure_1_ioaf139.pdf]

A

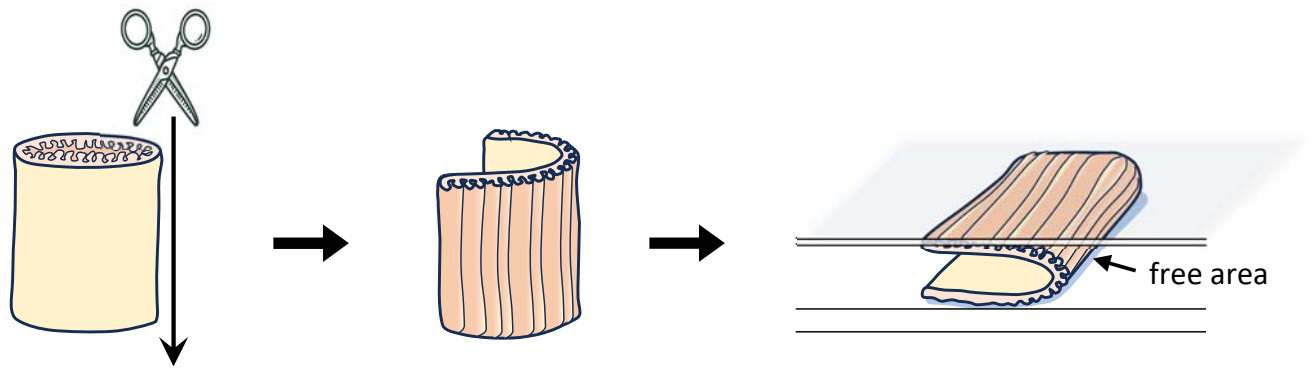

B

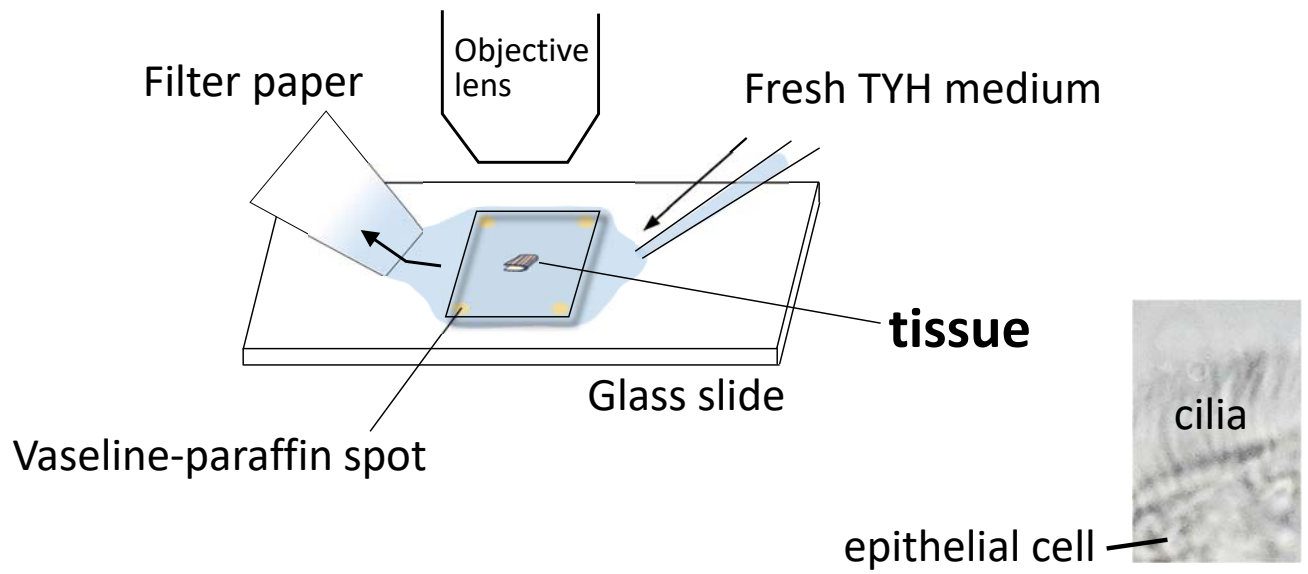

Supplement: Supplemental_Figure_2_ioaf139 [file supplemental_figure_2_ioaf139.pdf]

A

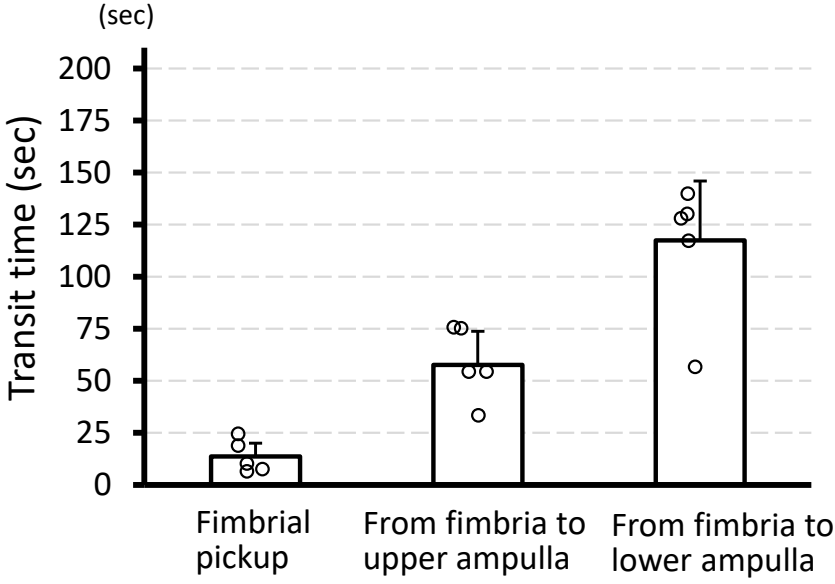

B

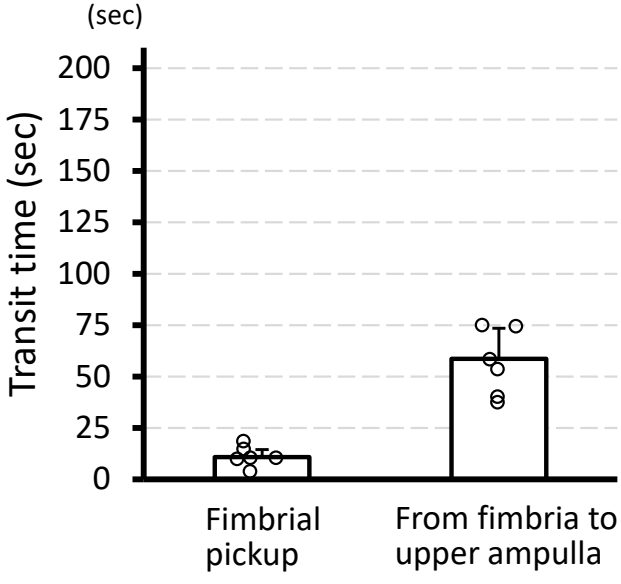

Supplement: Supplemental_Figure_3_ioaf139 [file supplemental_figure_3_ioaf139.pdf]
